# Supplementary material for: Hedonic processing in humans is mediated by an opioidergic mechanism in a mesocorticolimbic system
Source: eLife. 2018 Nov 16;7:e39648. doi: 10.7554/eLife.39648 (PMC6239433; doi:10.7554/eLife.39648)
Supplement: Supplementary file 1. — Two sided paired Wilcoxon signed rank test comparing mood between naloxone (_nlx) and saline (_nacl) sessions. [file elife-39648-supp1.docx]

|  | | | | | | | | | |
| --- | --- | --- | --- | --- | --- | --- | --- | --- | --- |
|  | |  | |  | | **W** | | **p** | |
| dry_mouth_nacl |  | - |  | dry_mouth_nlx |  | 13.500 |  | 0.530 |  |
| dry_skin_nacl |  | - |  | dry_skin_nlx |  | 13.500 |  | 0.530 |  |
| blurred_vision_nacl |  | - |  | blurred_vision_nlx |  | 4.000 |  | 0.773 |  |
| lethargy_nacl |  | - |  | lethargy_nlx |  | 10.000 |  | 0.110 |  |
| sickness_nacl |  | - |  | sickness_nlx |  | 22.500 |  | 0.530 |  |
| dizziness_nacl |  | - |  | dizziness_nlx |  | 9.000 |  | 0.766 |  |
| headache_nacl |  | - |  | headache_nlx |  | 36.000 |  | 0.802 |  |
