# Supplementary material for: Hedonic processing in humans is mediated by an opioidergic mechanism in a mesocorticolimbic system
Source: eLife. 2018 Nov 16;7:e39648. doi: 10.7554/eLife.39648 (PMC6239433; doi:10.7554/eLife.39648)
Supplement: Supplementary file 2. — Two sided paired Wilcoxon signed rank test comparing mood between naloxone (_nlx) and saline (_nacl) sessions. [file elife-39648-supp2.docx]

|  | |  | |  | | **W** | | **p** | |
| --- | --- | --- | --- | --- | --- | --- | --- | --- | --- |
| satisfied_nacl |  | - |  | satisfied_nlx |  | 12.00 |  | 0.407 |  |
| rested_nacl |  | - |  | rested_nlx |  | 32.50 |  | 0.594 |  |
| restless_nacl |  | - |  | restless_nlx |  | 45.50 |  | 0.594 |  |
| bad_nacl |  | - |  | bad_nlx |  | 12.00 |  | 0.824 |  |
| worn_out_nacl |  | - |  | worn_out_nlx |  | 32.50 |  | 0.594 |  |
| calm_nacl |  | - |  | calm_nlx |  | 33.00 |  | 0.198 |  |
| tired_nacl |  | - |  | tired_nlx |  | 82.50 |  | 0.789 |  |
| good_nacl |  | - |  | good_nlx |  | 30.00 |  | 0.260 |  |
| uneasy_nacl |  | - |  | uneasy_nlx |  | 67.00 |  | 0.115 |  |
| cheerful_nacl |  | - |  | cheerful_nlx |  | 64.00 |  | 0.841 |  |
| unwell_nacl |  | - |  | unwell_nlx |  | 76.00 |  | 0.684 |  |
| relaxed_nacl |  | - |  | relaxed_nlx |  | 37.50 |  | 0.301 |  |
