# Supplementary material for: Hedonic processing in humans is mediated by an opioidergic mechanism in a mesocorticolimbic system
Source: eLife. 2018 Nov 16;7:e39648. doi: 10.7554/eLife.39648 (PMC6239433; doi:10.7554/eLife.39648)
Supplement: Supplementary file 3. [file elife-39648-supp3.docx]

|  | | | |
| --- | --- | --- | --- |
|  |  | T(18) | p |
| Pleasure: High erotic > Low erotic | | 7.74 | p < 0.00001** |
|  |  |  |  |
| Frustration: High erotic > Low erotic | | 4.43 | p = 0.0002** |
|  |  |  |  |
|  |  |  |  |
| Pleasure: High money > Low money | | 7.19 | p < 0.00001** |
|  |  |  |  |
| Frustration: High money > Low money | | 6.90 | p < 0.00001** |
|  |  |  |  |
| * Significant at uncorrected threshold of p ≤ 0.05 (n=19, t-test)  ** Significant at corrected threshold of p ≤ 0.0125 (n=19, t-test corrected for 4 comparisons) | | | |
