# Supplementary material for: Hedonic processing in humans is mediated by an opioidergic mechanism in a mesocorticolimbic system
Source: eLife. 2018 Nov 16;7:e39648. doi: 10.7554/eLife.39648 (PMC6239433; doi:10.7554/eLife.39648)
Supplement: Supplementary file 4. — Repeated measures ANOVA, Huynh-Feldt nonsphericity correction. [file elife-39648-supp4.docx]

| **Within Subjects Effects** | | | | | | | | | | | |
| --- | --- | --- | --- | --- | --- | --- | --- | --- | --- | --- | --- |
|  |  |  | | **df** | | **Mean Square** | | **F** | | **p** | |
| Teatment |  |  |  | 1.000 |  | 637.59 |  | 4.063 |  | 0.060 |  |
| Teatment ✻ day_nlx |  |  |  | 1.000 |  | 230.60 |  | 1.469 |  | 0.242 |  |
| Condition |  |  | ᵃ | 3.948 | ᵃ | 13288.02 | ᵃ | 16.709 | ᵃ | < .001 | ᵃ |
| Condition ✻ day_nlx |  |  | ᵃ | 3.948 | ᵃ | 1333.01 | ᵃ | 1.676 | ᵃ | 0.167 | ᵃ |
| Teatment ✻ Condition |  |  | ᵃ | 5.217 | ᵃ | 105.77 | ᵃ | 2.598 | ᵃ | 0.029 | ᵃ |
| Teatment ✻ Condition ✻ day_nlx |  |  | ᵃ | 5.217 | ᵃ | 66.60 | ᵃ | 1.636 | ᵃ | 0.156 | ᵃ |

| **Between Subjects Effects** | | | | | | | | | | | |
| --- | --- | --- | --- | --- | --- | --- | --- | --- | --- | --- | --- |
|  | | **Sum of Squares** | | **df** | | **Mean Square** | | **F** | | **p** | |
| day_nlx |  | 6349 |  | 1 |  | 6349 |  | 2.778 |  | 0.114 |  |
