# Supplementary material for: Hedonic processing in humans is mediated by an opioidergic mechanism in a mesocorticolimbic system
Source: eLife. 2018 Nov 16;7:e39648. doi: 10.7554/eLife.39648 (PMC6239433; doi:10.7554/eLife.39648)
Supplement: Supplementary file 5. [file elife-39648-supp5.docx]

|  | | | |
| --- | --- | --- | --- |
| Reward |  | T(18) | p |
| Low money |  | -1.33 | 0.099 |
| Low money frustration |  | 0.10 | n/a |
| High money |  | -2.14 | 0.023* |
| High money frustration |  | -1.08 | 0.147 |
| Low erotic |  | -0.88 | 0.195 |
| Low erotic frustration |  | -0.12 | 0.454 |
| High erotic |  | -3.90 | 0.001”” |
| High erotic frustration |  | -2.80 | 0.006”” |
| * Significant at uncorrected threshold of p ≤ 0.05 (n=19, t-test)  ** Significant at corrected threshold of p ≤ 0.0063 (n=19, t-test corrected for 8 comparisons) | | | |
|  |  |  |  |
| Interaction |  |  |  |
| Low erotic > Low money |  | -0.29 | 0.39 |
| Low erotic frustration> Low money frustration |  | -1.54 | 0.07 |
| High erotic > High money |  | -1.66 | 0.057 |
| High erotic frustration> High money frustration |  | -2.61 | 0.009** |
| * Significant at uncorrected threshold of p ≤ 0.05 (n=19, t-test)  ** Significant at corrected threshold of p ≤ 0.0125 (n=19, t-test corrected for 4 comparisons) | | | |
|  |  |  |  |
