# Supplementary material for: Hedonic processing in humans is mediated by an opioidergic mechanism in a mesocorticolimbic system
Source: eLife. 2018 Nov 16;7:e39648. doi: 10.7554/eLife.39648 (PMC6239433; doi:10.7554/eLife.39648)
Supplement: Supplementary file 6. [file elife-39648-supp6.docx]

|  | | |
| --- | --- | --- |
| Ventral striatum L xyz(mm)  -12 9 -9 [4]  -12 12 -6 [5]  -9 8 -6 [1]  -9 9 -5 [1]  -12 12 -6 [2]  -6 10 0 [3]  **mean: -10 10 -5** | Ventral striatum R xyz(mm)  9 6 -9 [4]  12 3 -9 [5]  12 10 -6 [2]  8 10 2 [3]  **mean: 10 7 -6** | mPFC xyz(mm)  -6 45 -15 [4]  -6 24 36 [5]  6 24 -3 [5]  6 46 -15 [1]  0 39 5 [1]  3 44 -11 [1]  0 44 6 [1]  0 56 0 [2]  0 48 -2 [2]  0 56 -14 [2]  4 42 -16 [2]  0 32 -14 [2]  6 20 30 [2]  -2 34 10 [3]  2 48 14 [3]  6 30 -10 [3]  **mean: 1 40 0** |
| latOFC-L xyz(mm)  -30 33 -15 [4]  -30 33 -15 [5]  -30 22 0 [2]  -38 20 -12 [2]  -42 28 -6 [2]  -40 34 8 [2]  -44 38 -2 [2]  -46 32 0 [2]  -40 -6 6 [3]  -36 18 -2 [3]  -34 20 10 [3]  -26 30 -8 [3]  **mean: -36 25 -3** | latOFC R xyz(mm)  30 33 -15 [4]  30 33 -15 [5]  34 18 -18 [2]  32 30 -18 [2]  42 -6 6 [3]  28 26 -4 [3]  44 2 28 [3]  **mean: 34 19 -5** | Hypothalamus xyz(mm)  -4 -4 -6 [3]  -1 -4 -10 [1]  0 -8 -6 [7]  6 -6 -12 [6]  1 -7 -9 [8]  **mean: 0 -6 -8** |
| Amygdala L xyz(mm)  -21 -6 -27 [4]  -17 -3 -21 [1]  -16 -2 -14 [2]  -22 -4 -16 [3]  **mean: -19 -4 -20** | Amygdala R xyz(mm)  24 0 -24 [4]  20 -2 -16 [2]  20 -2 -14 [3]  **mean: 21 -1 -18** |  |

1. Kühn, S., and Gallinat, J. (2012). The neural correlates of subjective pleasantness. NeuroImage *61*, 289–294.

2. Morelli, S.A., Sacchet, M.D., and Zaki, J. (2015). Common and distinct neural correlates of personal and vicarious reward: A quantitative meta-analysis. NeuroImage *112*, 244–253.

3. Noori, H.R., Cosa Linan, A., and Spanagel, R. (2016). Largely overlapping neuronal substrates of reactivity to drug, gambling, food and sexual cues: A comprehensive meta-analysis. Eur. Neuropsychopharmacol. J. Eur. Coll. Neuropsychopharmacol. *26*, 1419–1430.

4. Sescousse, G., Redouté, J., and Dreher, J.-C. (2010). The architecture of reward value coding in the human orbitofrontal cortex. J. Neurosci. Off. J. Soc. Neurosci. *30*, 13095–13104.

5. Sescousse, G., Barbalat, G., Domenech, P., and Dreher, J.-C. (2013). Imbalance in the sensitivity to different types of rewards in pathological gambling. Brain J. Neurol. *136*, 2527–2538.

6. Arnow, B.A., Desmond, J.E., Banner, L.L., Glover, G.H., Solomon, A., Polan, M.L., Lue, T.F., and Atlas, S.W. (2002). Brain activation and sexual arousal in healthy, heterosexual males. Brain *125*, 1014–1023.

7. Redouté, J., Stoléru, S., Grégoire, M.C., Costes, N., Cinotti, L., Lavenne, F., Le Bars, D., Forest, M.G., and Pujol, J.F. (2000). Brain processing of visual sexual stimuli in human males. Hum. Brain Mapp. *11*, 162–177.

8. Beauregard, M., Lévesque, J., and Bourgouin, P. (2001). Neural correlates of conscious self-regulation of emotion. J. Neurosci. Off. J. Soc. Neurosci. *21*, RC165.
