# Supplementary material for: Hedonic processing in humans is mediated by an opioidergic mechanism in a mesocorticolimbic system
Source: eLife. 2018 Nov 16;7:e39648. doi: 10.7554/eLife.39648 (PMC6239433; doi:10.7554/eLife.39648)
Supplement: Supplementary file 7. [file elife-39648-supp7.docx]

|  | | | |
| --- | --- | --- | --- |
| Region of Interest (ROI) | Right/Left | T(18) | P |
|  |  |  |  |
| Ventral Striatum | R | -2.99 | 0.004** |
|  | L | -3.47 | 0.001** |
|  |  |  |  |
| Lateral OFC | R | -2.84 | 0.006** |
|  | L | -1.94 | 0.034* |
|  |  |  |  |
| Amygdala | R | -2.85 | 0.005** |
|  | L | -2.83 | 0.006** |
|  |  |  |  |
| Medial Prefrontal Cortex |  | -3.52 | 0.001** |
|  |  |  |  |
| Hypothalamus |  | -1.87 | 0.04* |
|  |  |  |  |
| * Significant at uncorrected threshold of p ≤ 0.05 (n=19, t-test)  ** Significant at corrected threshold of p ≤ 0.0063 (n=19, t-test corrected for 8 ROIs) | | | |
