# Supplementary material for: Hedonic processing in humans is mediated by an opioidergic mechanism in a mesocorticolimbic system
Source: eLife. 2018 Nov 16;7:e39648. doi: 10.7554/eLife.39648 (PMC6239433; doi:10.7554/eLife.39648)
Supplement: Supplementary file 8. [file elife-39648-supp8.docx]

|  | | | |
| --- | --- | --- | --- |
| Region of Interest (ROI) | Right/Left | T(18) | P |
|  |  |  |  |
| Ventral Striatum | R | 1.18 | 0.126 |
|  | L | 2.56 | 0.0099* |
|  |  |  |  |
| Lateral OFC | R | 2.40 | 0.0136* |
|  | L | 1.39 | 0.091 |
|  |  |  |  |
| Amygdala | R | 3.40 | 0.0016** |
|  | L | 3.50 | 0.0013** |
|  |  |  |  |
| Medial Prefrontal Cortex |  | 2.23 | 0.0195* |
|  |  |  |  |
| Hypothalamus |  | 1.54 | 0.071 |
|  |  |  |  |
| * Significant at uncorrected threshold of p ≤ 0.05 (n=19, t-test)  ** Significant at corrected threshold of p ≤ 0.0063 (n=19, t-test corrected for 8 ROIs) | | | |
