# Supplementary material for: Hedonic processing in humans is mediated by an opioidergic mechanism in a mesocorticolimbic system
Source: eLife. 2018 Nov 16;7:e39648. doi: 10.7554/eLife.39648 (PMC6239433; doi:10.7554/eLife.39648)
Supplement: Supplementary file 9. [file elife-39648-supp9.docx]

|  | | | |
| --- | --- | --- | --- |
| **Anticipation for high > low erotic pictures** | | | |
|  | | | |
| Region of Interest (ROI) | Right/Left | T(18) | P (uncorrected) |
|  |  |  |  |
| Ventral Striatum | R | -0.63 | 0.27 |
|  | L | -0.66 | 0.26 |
|  |  |  |  |
| Lateral OFC | R | -0.33 | 0.37 |
|  | L | -0.63 | 0.27 |
|  |  |  |  |
| Amygdala | R | -0.53 | 0.30 |
|  | L | -0.79 | 0.22 |
|  |  |  |  |
| Medial Prefrontal Cortex |  | 0.22 | n/a |
|  |  |  |  |
| Hypothalamus |  | -1.45 | 0.082 |
|  |  |  |  |
| **Anticipation for high > low monetary rewards** | | | |
|  | | | |
| Region of Interest (ROI) | Right/Left | T(18) | P |
|  |  |  |  |
| Ventral Striatum | R | -0.21 | 0.42 |
|  | L | -1.04 | 0.16 |
|  |  |  |  |
| Lateral OFC | R | -1.82 | 0.04* |
|  | L | -1.60 | 0.06 |
|  |  |  |  |
| Amygdala | R | 0.39 | n/a |
|  | L | 0.23 | n/a |
|  |  |  |  |
| Medial Prefrontal Cortex |  | -1.90 | 0.04* |
|  |  |  |  |
| Hypothalamus |  | -1.54 | 0.07 |
|  |  |  |  |
| **Outcome for high > low monetary rewards** | | | |
|  | | | |
| Region of Interest (ROI) | Right/Left | T(18) | P (uncorrected) |
|  |  |  |  |
| Ventral Striatum | R | -0.51 | 0.31 |
|  | L | 0.14 | n/a |
|  |  |  |  |
| Lateral OFC | R | 0.26 | n/a |
|  | L | -0.28 | 0.39 |
|  |  |  |  |
| Amygdala | R | 0.49 | n/a |
|  | L | 1.19 | n/a |
|  |  |  |  |
| Medial Prefrontal Cortex |  | -0.24 | 0.41 |
|  |  |  |  |
| Hypothalamus |  | 0.20 | n/a |
|  |  |  |  |
| * Significant at uncorrected threshold of p ≤ 0.05 (n=19, t-test)  ** Significant at corrected threshold of p ≤ 0.0063 (n=19, t-test corrected for 8 ROIs) | | | |
