# Supplementary material for: Hedonic processing in humans is mediated by an opioidergic mechanism in a mesocorticolimbic system
Source: eLife. 2018 Nov 16;7:e39648. doi: 10.7554/eLife.39648 (PMC6239433; doi:10.7554/eLife.39648)
Supplement: Supplementary file 10. [file elife-39648-supp10.docx]

|  | | | |
| --- | --- | --- | --- |
| Region of Interest (ROI) | Right/Left | T(17) | P |
|  |  |  |  |
| Ventral Striatum | R | 0.95 | 0.18 |
|  | L | 2.09 | 0.03* |
|  |  |  |  |
| Lateral OFC | R | 1.39 | 0.09 |
|  | L | 0.59 | 0.28 |
|  |  |  |  |
| Amygdala | R | 1.06 | 0.15 |
|  | L | 0.70 | 0.25 |
|  |  |  |  |
| Medial Prefrontal Cortex |  | 0.23 | 0.41 |
|  |  |  |  |
| Hypothalamus |  | 3.24 | 0.002*** |
|  |  |  |  |
| * Significant at uncorrected threshold of p ≤ 0.05 (n=19, t-test)  ** Significant at corrected threshold of p ≤ 0.0063 (n=19, t-test corrected for 8 ROIs) | | | |
